# Frontiers Commentary on Tallet et al. Investigation of Prolactin Receptor Activation and Blockade Using Time-Resolved Fluorescence Resonance Energy Transfer

**DOI:** 10.3389/fendo.2014.00088

**Published:** 2014-06-18

**Authors:** Ralf Jockers

**Affiliations:** ^1^INSERM, U1016, Institut Cochin, Paris, France; ^2^CNRS UMR 8104, Paris, France; ^3^University Paris Descartes, Sorbonne Paris Cité, Paris, France

**Keywords:** dimerization, TR-FRET, PRLR-I146L, antagonist, commentary

Transmission of extracellular signals into the cell often relies on the binding of extracellular signaling molecules to membrane receptors. How membrane receptors transmit these signals into the cell and activate intracellular signaling cascades has been an intense field of research over the last 20 years. Basically, two competing models have been proposed. In the first model, binding of signaling molecules promotes ligand-induced dimerization/oligomerization of receptors. According to the second model, binding of signaling molecules triggers ligand-induced conformational changes within preformed dimeric/oligomeric receptors.

For many years, the model of ligand-induced dimerization was the preferred model to explain the activation of many single transmembrane-spanning receptors that rely on the (trans)phosphorylation of their intracellular domains. One of these receptors is the prolactin receptor (PRLR), which belongs to the cytokine receptor family that is devoid of intrinsic kinase activity but constitutively interacts with the JAK2 tyrosine kinase. PRLR activation triggers first trans-phosphorylation of two JAK2 molecules (each bound to one PRLR molecule), followed by receptor phosphorylation by JAK2 and recruitment of STAT5 molecules promoting further downstream signaling events.

Before the publication of the article of Tallet et al. in *Frontiers in Endocrinology* (1), conflicting results had been reported in the literature concerning the activation model of the PRLR. Indeed, Qazi et al., using bioluminescence resonance energy transfer (BRET) and co-immunoprecipitation (co-IP) approaches, concluded that the PRLR constitutively homodimerize (or heterodimerized when long and short isoforms were co-expressed in the same cell) (2). A similar conclusion was reached in another study based on co-IP experiments further arguing for the existence of ligand-independent homodimers of human PRLR isoforms, with the transmembrane domain being the main dimer interface (3). Contrasting results were however reported in another BRET study with various C-terminally tagged PRLR isoforms. The authors revealed ligand-dependent changes in BRET signals that discriminated receptor agonists from a partial agonist/antagonist (4). Unfortunately, the authors did not perform complementary experiments to determine whether the BRET changes originated from ligand-induced dimerization or conformational changes as previously reported for other cytokine receptors (5).

The article of Tallet et al. published in *Frontiers in Endocrinology* made a decisive contribution because the authors recapitulated all previously employed techniques (co-IP, BRET) in a single study and went beyond the current status by exploiting further techniques such as blue native gel electrophoreses and time-resolved fluorescence resonance energy transfer (TR-FRET). This presents the first application of the TR-FRET technology to cytokine receptors. TR-FRET is based on the energy transfer between the energy donor terbium cryptate (Tb) and an energy acceptor. Due to the long-lived fluorescence properties of Tb, measurement can be performed after a time delay of 50–150 μs to avoid interference with cellular autofluorescence, thereby considerably improving the signal-to-noise ratio of the assay (6). TR-FRET has several interesting features that differentiate this technique from BRET. Whereas BRET signals typically represent all tagged proteins that interact, irrespective of their subcellular localization, TR-FRET signals are only generated by the fraction of proteins located at the plasma membrane. This difference seems to be important as the PRLR is mainly located inside the cell and only the fraction that reaches the cell surface becomes activated by the extracellular prolactin. Furthermore, the intensity of TR-FRET signals directly correlates with the distance between the donor and acceptor, which allows us to be conclusive regarding this parameter. This is more difficult to achieve with the BRET technique as BRET signals depend not only on the distance but also on the relative orientation of the BRET donor and acceptor. In the study of Tallet et al., the authors designed a TR-FRET assay to monitor putative ligand-induced TR-FRET signal changes between two extracellular PRLR domains. Taken all techniques together, the authors convincingly demonstrated that (at least part of) the PRLR population is pre-dimerized at the cell membrane and that activation (via ligand binding or I146L mutation that constitutively activates the receptor) or inactivation (by receptor antagonists) do not alter the number of dimers or the distance between the two extracellular domains of PRLR dimers, suggesting that the control of receptor activity involves subtle conformational changes.

A subsequent study that solved the nuclear magnetic resonance solution structure of the membrane proximal D2 domain of the human PRLR (7), confirmed this conclusion as the authors showed that the conserved WSXWS motif of the D2 domain undergoes conformational changes upon ligand binding (from a T-stack to a ladder) which serves as a molecular switch for activation. Consistently, the constitutively active PRLR-I146L showed a change in the dynamic of this motif.

The article of Tallet et al. had an important impact in its field as it allowed to exclude one hypothesis and definitely oriented the search for intramolecular conformational changes without impact on receptor stoichiometry or major changes in the distance of the extracellular domains. Future work will now fully concentrate on the mechanism of PRLR activation involving intramolecular conformational changes. Recent advances on the activation mechanism of other cytokine receptors such as the growth hormone receptor (GHR) appear to consolidate the proposed PRLR activation model and is likely to provide additional insights (8). According to the current model, GHR exists as preformed inactive dimer in which the two subunits are held together through weak interactions in the transmembrane domain. Receptor dimers are proposed to associate with a JAK2 dimer through the Box 1 motif in the intracellular region of the receptor in a way that the kinase domain of one JAK2 molecule binds to the pseudokinase domain of the other JAK2 molecule in “trans,” thus inhibiting kinase activity in the basal state. Binding of growth hormone to GHR alters the position of the extracellular juxtamembrane, transmembrane, and intracellular domain of the GHR in an outward movement aligning and activating the two JAK2 kinase domains. Future studies will show whether PRLR activation fits to this model. Such a mechanism opens also new therapeutic routes. For example, antibodies directed against the extracellular juxtamembrane domain might stabilize the active conformation of the receptor or disruption of the JAK2 kinase/pseudokinase interaction with small molecules might help to activate the kinase.

## Conflict of Interest Statement

The author declares that the research was conducted in the absence of any commercial or financial relationships that could be construed as a potential conflict of interest.
